# MR imaging tracking of inflammation-activatable engineered neutrophils for targeted therapy of surgically treated glioma

**DOI:** 10.1038/s41467-018-07250-6

**Published:** 2018-11-14

**Authors:** Meiying Wu, Haixian Zhang, Changjun Tie, Chunhong Yan, Zhiting Deng, Qian Wan, Xin Liu, Fei Yan, Hairong Zheng

**Affiliations:** 1 Paul C. Lauterbur Research Center for Biomedical Imaging, Institute of Biomedical and Health Engineering, Shenzhen Institutes of Advanced Technology, Chinese Academy of Sciences, Shenzhen, 518055 P.R. China; 2 Department of Ultrasound, Fudan University Shanghai Cancer Center, Shanghai, 200032 P.R. China; 3 Shenzhen Hospital of Guangzhou University of Chinese Medicine, Shenzhen, 518034 P.R. China

## Abstract

Cell-based drug delivery systems have shown promising capability for tumor-targeted therapy owing to the intrinsic tumor-homing and drug-carrying property of some living cells. However, imaging tracking of their migration and bio-effects is urgently needed for clinical application, especially for glioma. Here, we report the inflammation-activatable engineered neutrophils by internalizing doxorubicin-loaded magnetic mesoporous silica nanoparticles (ND-MMSNs) which can provide the potential for magnetic resonance (MR) imaging tracking of the drug-loaded cells to actively target inflamed brain tumor after surgical resection of primary tumor. The phagocytized D-MMSNs possess high drug loading efficiency and do not affect the host neutrophils’ viability, thus remarkably improving intratumoral drug concentration and delaying relapse of surgically treated glioma. Our study offers a new strategy in targeted cancer theranostics through combining the merits of living cells and nanoparticle carriers.

## Abstract (web summary)

Imaging tracking of the migration of cell-based drug delivery systems are needed for expanding their clinical application for glioma. Here they report inflammation activatable engineered neutrophils containing doxorubicin-loaded magnetic mesoporous silica nanoparticles to image and actively target brain tumors after resection.

## Introduction

Glioma has been considered to be the most frequent primary central nervous system tumor with poor prognosis, high recurrence, and mortality rate^1–3^. Conventional surgical resection, a first-line treatment method for the patients with glioma, achieves limited clinical therapeutic outcome due to the highly infiltrative and invasive nature of glioma cells^4–6^. Generally, adjuvant chemotherapy after surgery is required, but its effect is hindered by the limited drug penetration through the various physiological barriers, especially the blood−brain barrier (BBB) and blood−tumor barrier (BTB)^7,8^. Considerable efforts have been focused on the nanoparticle-based drug delivery systems (NDDSs) to traverse BBB/BTB by using active targeting ligands or passive leakage of tumor vasculature^9–12^. However, the therapeutic efficacy of NDDSs is still unsatisfied due to poor blood circulation lifetime of nanoparticles, insufficient intratumoral drug accumulation, and severe systematic toxicity.

In the past decade, cell-based drug delivery systems (CDDSs) have been increasingly recognized as bioinspired and powerful drug delivery platforms for glioma treatment^13–17^. Mesenchymal stem cells (MSCs) and neural stem cells (NSCs) were proven to possess intrinsic tumor-homing capacity, allowing them to deliver therapeutic agents to malignant and invasive glioma foci^18–21^. For instance, Yan et al. recently demonstrated that MSCs could be transduced with nonviral vectors to express human tumor necrosis factor-related apoptosis-inducing ligand for inducing apoptosis of glioma cells, without affecting their natural proliferation, differentiation and tumor-specific homing capabilities^20^. Lesniak also reported nanoparticle-programmed self-destructive NSCs were capable of dispersing throughout the tumor mass after contralateral injection of tumor due to the tumor-tropic migratory capacity of NSCs^21^. These studies suggest that MSCs/NSCs-based CDDSs would be an attractive approach for tumor-targeted drug/gene delivery.

Recently, immune cells have attracted intensive attention as “living” drug delivery vehicles because they can travel through blood flow and migrate to sites of injury, inflammation or tumor with reduced immune clearance and prolonged biological half-life^22–26^. Neutrophils, a type of polymorphonuclear leukocyte, play a critical role in immune responses. They can be activated within the vasculature and move along the chemotactic gradients towards the inflammatory sites, and eliminate the pathogens by phagocytosis^27–29^. In addition, they possess the native ability of crossing BBB/BTB and infiltrating the tumor mass^30–33^, thereby being explored as “Trojan horses” to carry concealed drug cargoes to diseased brain areas. Zhang et al. demonstrated that neutrophils carrying paclitaxel-liposomes still maintained the physiological activities of neutrophils and migrated to the inflamed brain tumor, resulting in improved survival time of postsurgical glioma-bearing mice^34^. However, this study mainly focuses on the therapeutic efficacy of neutrophil-based CDDSs. The location and behavior of neutrophils after internalizing DDSs within the body still remain unclear.

**Fig. 1:**
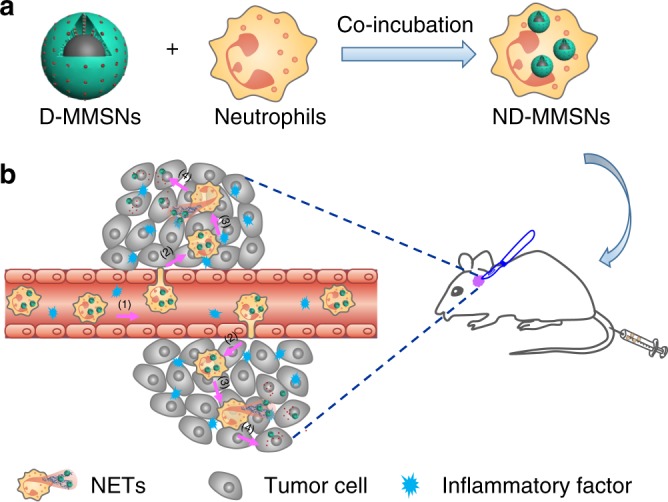
Fabrication and targeted-therapeutic schematics of ND-MMSNs. **a** Schematic illustration of the preparation of ND-MMSNs. **b** Schematic shows that inflammation-activatable ND-MMSNs target inflamed glioma sites and phagocytized D-MMSNs would be released to achieve residual tumor theranostics

**Fig. 2:**
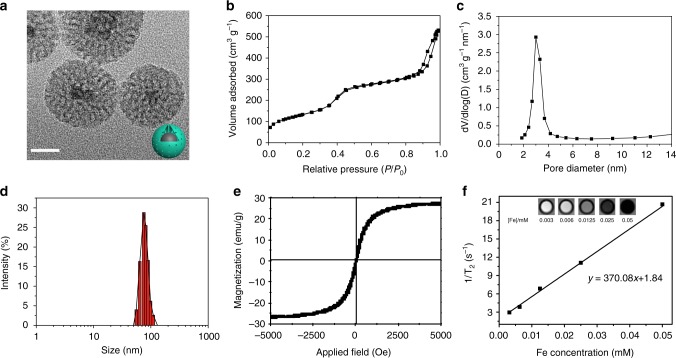
Structural and compositional characterizations of MMSNs. **a** TEM image of MMSNs. Scale bar: 20 nm. **b** N_2_ adsorption−desorption isotherm and **c** corresponding pore-size distribution of MMSNs. **d** The hydrodynamic particle-size distribution of MMSNs in aqueous solution by DLS measurement. **e** Field-dependent magnetization curve of MMSNs measured at room temperature. **f** Plots of T_2_^−1^ versus Fe concentration and concentration-dependent T_2_-weighted MR imaging for MMSNs solution. Transverse relaxivity (*r*_2_) was derived from linear fitting of the plots

**Fig. 3:**
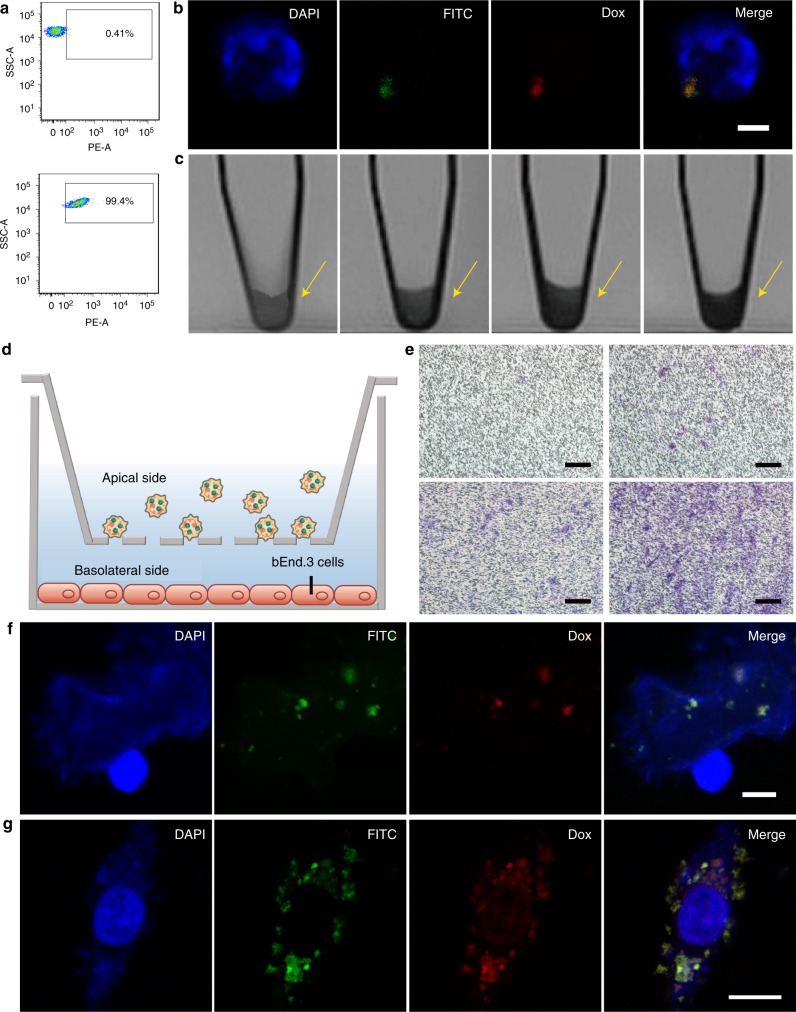
Isolation and characterization of neutrophils. **a** Flow cytometry data of neutrophils treated with culture media and D-MMSNs for 1 h, respectively. Dox fluorescence signal was monitored at 488 nm via the PE-A channel. **b** CLSM images of neutrophils after coincubation with D-FMMSNs for 1 h ([Dox] = 10 μg ml^−1^). Blue fluorescence represents the polymorphonuclear of neutrophils stained with DAPI. Green fluorescence represents FITC-labeled MMSNs. Red fluorescence represents Dox encapsulated in the D-MMSNs. Scale bar: 2.5 μm. **c** T_2_-weighted MRI of neutrophils after coincubation with D-MMSNs of varied concentrations (from left to right: 0, 12.5, 25, and 50 μg ml^−1^, respectively) for 1 h. **d** Schematic illustration of in vitro inflammation response evaluation. **e** Representative images of migration of ND-MMSNs towards fresh serum-free media and fresh serum-free media + TNF-α for 30 min, 1, and 2 h. Scale bar: 100 μm. **f** CLSM images of NETs after ND-FMMSNs incubated with U87 glioma cells for 2 h. Neutrophil-derived DNA networks were stained with DAPI emitting blue fluorescence. Green fluorescence: FITC-labeled MMSNs. Red fluorescence: Dox. Scale bar: 5 μm. **g** CLSM images of U87 glioma cells after incubation with ND-FMMSNs for 4 h ([Dox] = 10 μg ml^−^^1^). After incubation, U87 cell nuclei were stained with DAPI, which emits blue fluorescence. Green fluorescence: FITC-labeled MMSNs. Red fluorescence: Dox. Scale bar: 10 μm

**Fig. 4:**
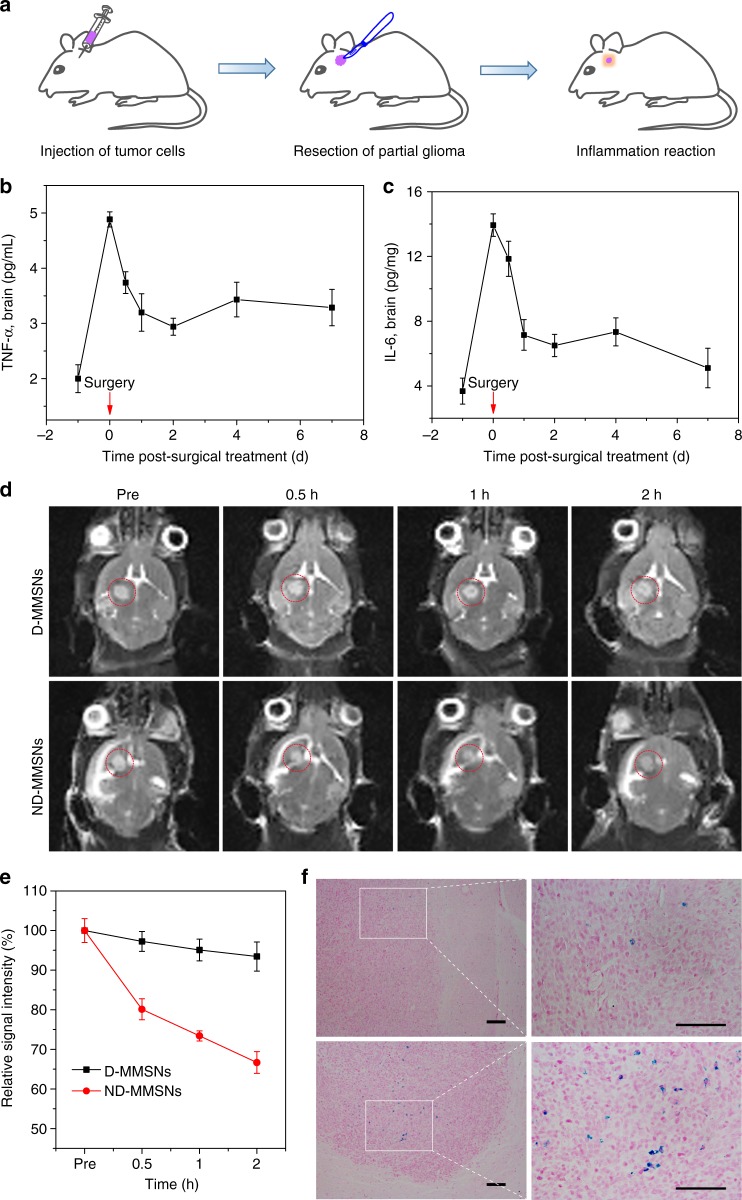
In vivo T_2_-weighted MR imaging. **a** Schematic representation of in vivo inflammation reaction after resecting partial glioma. Evaluation of the inflammation cytokines TNF-α (**b**) and IL-6 (**c**) levels in the brain of the U87-bearing mice after surgical treatment for 7 d. **d** In vivo T_2_-weighted MR images of postsurgical glioma-bearing mice before and after intravenous injection of D-MMSNs and ND-MMSNs. The red circles point to the tumor site. **e** Relative signal intensities of postsurgical glioma as a function of time. **f** Histological analysis of brain tissues by Prussian blue staining. Scale bar: 50 μm. Mean values and error bars are defined as mean and s.d., respectively (*n* = 3)

Magnetic resonance imaging (MRI) technique has been extensively studied for cell tracking because of its noninvasiveness, high spatial resolution, deep penetration depth, and relatively long retention of MRI contrast agents in cells^35–38^. Nevertheless, the properties of neutrophils after labeling with MRI contrast agents and their subsequent bio-effects have not been explored and studied. It is worth noting that neutrophils are phagocytic cells and can uptake various nanoparticles^39,40^. In addition, neutrophils are terminal-differentiated cells with an average half-life of 6−7 h^41,42^; thus, the MRI signal would not decrease due to cell proliferation and cellular exocytosis. Therefore, in this study, we explore core-shell structured magnetic mesoporous silica nanoparticles (designated as MMSNs) as neutrophils tracking probes and drug delivery nanocarriers for inflamed glioma-targeted theranostics (Fig. 1). The nanoparticles combine the merits of magnetic Fe_3_O_4_ core that provides contrast enhancement for MRI, and mesoporous silica shell for the encapsulation/sustained release of chemotherapeutic agents. Doxorubicin (Dox), a model antitumor drug, was loaded into MMSNs (designated as D-MMSNs) and coincubated with neutrophils from peripheral blood of health mice, thus producing an intelligent biomimetic theranostic platform ND-MMSNs (Fig. 1a). After systemic injection of ND-MMSNs into the inflamed mouse glioma model established by surgically resecting part of primary glioma, they can migrate along the molecular guidance signals (chemoattractants or chemokines) (Fig. 1b–1), emigrate outside the vasculature and accumulate in the inflamed glioma sites (Fig. 1b, 2), resulting in the inflammation-triggered neutrophil recruitment^40,43^. Subsequently, highly activated neutrophils carrying D-MMSNs release neutrophil extracellular traps (NETs) in the inflammatory region (Fig. 1b–3)^44,45^. The concomitant release of D-MMSNs cargos are uptaken by infiltrating glioma cells, achieving precise diagnosis and high anti-glioma efficacy (Fig. 1b–4).

## Results

### Fabrication and characterization of MMSNs

MMSNs were synthesized by encapsulating hydrophobic magnetic Fe_3_O_4_ nanoparticles in mesoporous silica spheres, in which cetyltrimethylammonium bromide (CTAB) surfactant not only as a phase transfer agent but also as a porogen for the formation of mesostructure^46^. The well-defined morphology and core/shell structure of MMSNs can be clearly observed from the contrast difference in the transmission electron microscopy (TEM) image (Fig. 2a). The existence of Si, O, and Fe elements in the MMSNs was verified by the energy dispersive X-ray spectrum (Supplementary Fig. 1a). Meanwhile, the element mapping further indicates that mesoporous silica has been successfully coated onto the Fe_3_O_4_ inner core to form the core/shell structure (Supplementary Fig. 1b). MMSNs possess the well-defined mesoporous structure with a Brunauer−Emmett−Teller (BET) surface area of 485.25 m^2^ g^−1^, a pore volume of 0.85 cm^3^ g^−1^ and an average pore size of 3.0 nm demonstrated by N_2_ adsorption−desorption isotherm (Fig. 2b, c). Additionally, MMSNs present a narrow size distribution in aqueous solution with an average hydrated particle size of 75.3 nm, which was determined by dynamic light scattering (DLS, Fig. 2d). Notably, the field-dependent magnetization curve of MMSNs shows a negligible hysteresis loop with a magnetization saturation of 27.1 emu g^−1^, confirming the excellent superparamagnetism of MMSNs at room temperature (Fig. 2e and Supplementary Fig. 2). To assess the MRI capability of MMSNs, the transverse relaxivity was also measured using a clinical 3.0 T MRI scanner. As shown in Fig. 2f, the MMSNs solutions are observed to become darker in T_2_-weighted MR images as Fe concentration increases. The *r*_2_ relaxation value of MMSNs has been calculated to be 370.08 mM^−1^ s^−1^, suggesting their potential as T_2_-weighted MRI contrast agents^47,48^.

### Evaluation of ND-MMSNs functions

Neutrophils were isolated from peripheral blood of mice via the Percoll gradient method as previously described^49^. A typical polymorphonuclear morphology is observed under optical microscope after Wright−Giemsa staining (Supplementary Fig. 3a). Neutrophils are short-lived cells and can rapidly capture exogenous nanoparticles through phagocytosis; therefore, we firstly investigated the cytotoxicity of MMSNs against neutrophils. No obvious toxicities are seen under the tested concentrations (Supplementary Fig. 3b), suggesting the high biocompatibility of MMSNs. Here, Dox can be loaded into the mesoporous channels of MMSNs with high loading efficiency (94.5%), and one can see that the mass ratio of Si, Fe, and Dox is 1:0.81:3.45. Flow cytometry was then employed to assess the proportion of neutrophils that have phagocytized D-MMSNs. After 1 h of incubation, 99.4% of neutrophils show significant uptake of D-MMSNs (Fig. 3a), demonstrating the strong phagocytic capability of neutrophils. The resulting ND-MMSNs has a loading capacity of 4.97×10^−5^ µg Dox per cell and 1.62×10^−5^ µg Fe_3_O_4_ per cell. As we know, phagocytic vacuole is the main arena for neutrophil to kill and digest engulfed particles, and its acidification has been considered an important antimicrobial event^50–53^. Therefore, the iron- and Dox-releasing patterns of D-MMSNs were monitored under different pH buffer solutions (pH = 7.4, 6.0 or 5.0). No iron element could be released from the prepared D-MMSNs due to the mild acidic environment (Supplementary Fig. 4a). The pH-dependent and sustained Dox-releasing behaviors have been achieved when the pH value of buffer solution reduces from 7.4 to 5.0 (Supplementary Fig. 4b). To investigate the intracellular drug deposition and the effect of drug release on cell activity, a series of in vitro cell experiments were conducted. As shown in Fig. 3b, MMSNs labeled with fluorescein isothiocyanate (FITC) green fluorescence (FMMSNs) are colocalized with Dox red fluorescence, which could be observed in the peri-polymorphonuclear region of neutrophils in 1 h of phagocytosis, indicating the efficient loading of D-FMMSNs into neutrophils and the formation of ND-FMMSNs. The cell survival of ND-MMSNs was also evaluated by standard cell viability assay (Supplementary Fig. 5a) and live/dead co-stained assay (Supplementary Fig. 5b). The results demonstrate that the proportion of dead neutrophils increases with the prolonged incubation time when incubated with the same Dox concentration of D-MMSNs. Considering the high cellular uptake amounts and low cytotoxicity of neutrophils in 1 h of phagocytosis, we chose 1 h as the incubation time for the preparation of ND-MMSNs.

In vitro T_2_-weighted MRI of neutrophils was investigated by coincubation with different concentrations of D-MMSNs (0, 12.5, 25 and 50 μg ml^−1^, [Dox] = 10 μg ml^−1^) for 1 h. The neutrophils bearing D-MMSNs were centrifuged and precipitated at the bottom of the tubes. As shown in Fig. 3c, the MRI signal intensity increases with the enhanced concentrations of D-MMSNs, illustrating the successful internalization of D-MMSNs by neutrophils and the dose-dependent internalization behavior. To evaluate whether neutrophils maintain the chemotactic capability after phagocytosing D-MMSNs, a transwell membrane system with the chemical gradient of cytokines was established by placing tumor necrosis factor (TNF)-α-stimulated bEnd.3 endothelial cells in the basolateral side (Fig. 3d)^54,55^. Scarcely any neutrophils bearing D-MMSNs migrate through the transwell membrane pore when the subnatant well is filled with fresh serum-free media. By contrast, the number of neutrophils moving across the transwell membrane increases over time when the bEnd.3 endothelial cell monolayers were treated with fresh serum-free media containing TNF-α (Fig. 3e), indicating the chemotactic behavior of ND-MMSNs stimulated by proinflammatory cytokines. These results reveal that neutrophils internalizing D-MMSNs do not compromise inherent cell properties such as cell viability, migration and their ability of responding to the chemoattractant gradients.

To investigate the mechanism of how drugs are released from neutrophils to come into function, we cultured U87 or C6 glioma cells on the confocal dishes and incubated them with ND-FMMSNs. After coincubation for 2 or 4 h, ND-FMMSNs were gently removed by washing with phosphate buffer saline (PBS). As can be seen from Fig. 3f and Supplementary Fig. 6, D-FMMSNs are observed in the extracellular traps (ETs) from neutrophils (i.e., NETs) in 2 h of coincubation. Notably, red fluorescence can be well merged with FITC green fluorescence, suggesting Dox was kept in D-FMMSNs at that time. When coincubation time was extended to 4 h, it is found that Dox red fluorescence signals are largely present in the cytoplasm region and a few fluorescence signals in the nuclei of tumor cells. But all of MMSNs labeled with FITC green fluorescence appear in the cytoplasm region of tumor cells (Fig. 3g and Supplementary Fig. 7). These results demonstrate that D-MMSNs, along with NET formation, could be released from neutrophils and then uptaken by glioma cells. To determine whether exosomes participate in the delivery process of D-MMSNs from neutrophils to glioma cells, the exosomes released from ND-MMSNs were collected to analyze their contents. No nanoparticles are found in these vesicles by TEM technique (Supplementary Fig. 8a and b) and only few free Dox could be detected by high-performance liquid chromatography (Supplementary Fig. 8c−e). These results indicate chemotherapeutic drugs Dox are largely kept in D-MMSNs and released from neutrophils through NET formation but not through exosome secretion. Next, we further studied in vitro cytotoxicity against U87, C6 glioma cells and bEnd.3 endothelial cells by the typical CCK-8 assay (Supplementary Fig. 9a and b). The results reveal that neutrophils themselves exhibit negligible cytotoxicity to U87, C6 glioma cells and bEnd.3 endothelial cells, whereas neutrophils carrying D-MMSNs induce markedly decreased cell viabilities at elevated Dox concentration, indicating the internalized D-MMSNs by neutrophils play a key role in performing the anticancer efficacy while neutrophils themselves function as targeted cell carriers.

### In vivo neutrophil recruitment

To further evaluate the inflammation-triggered neutrophil recruitment to inflamed brain tumor, an incomplete resection of orthotopic glioma model was established (Fig. 4a). The expression levels of proinflammatory cytokines such as TNF-α and interleukin (IL)-6 in the inflamed brain (Fig. 4b, c) and the blood (Supplementary Fig. 10a and b) of postsurgical U87 glioma-bearing mice were measured using commercial enzyme-linked immunosorbent assay (ELISA) kits. The results show that the production level of TNF-α and IL-6 on the day of surgery is significantly higher than that before surgery, and decreases over time. It illustrates that surgery promotes the secretion of proinflammatory cytokines, which would facilitate inflammation-driven ND-MMSNs recruitment.

Subsequently, we investigated the capacity of ND-MMSNs homing to in vivo inflamed brain tumor via T_2_-weighted MR images of surgically treated glioma-bearing mice before and after intravenous administration of ND-MMSNs. Compared to D-MMSNs group, ND-MMSNs group shows strong contrast enhancement of negative signals in the postsurgical glioma region (Fig. 4d). The contrast enhancement effects could be further quantitatively measured by T_2_-weighted MR signal intensity. As shown in Fig. 4e, the relative signal intensity of ND-MMSNs decreases by more than 30% in 2 h owing to the chemotactic capability of neutrophils. The iron deposition in brain was further examined through histological analysis on brain sections by Prussian blue staining, revealing more nanoparticles accumulate in the residual glioma site in the ND-MMSNs group than that in the D-MMSNs group (Fig. 4f). It is consistent with the results of in vivo T_2_-weighted MRI performance. These results confirm D-MMSNs could be efficiently delivered into the inflamed brain tumor tissue by neutrophils, and used for in vivo neutrophil tracking by MRI.

### In vivo biodistribution

**Fig. 5:**
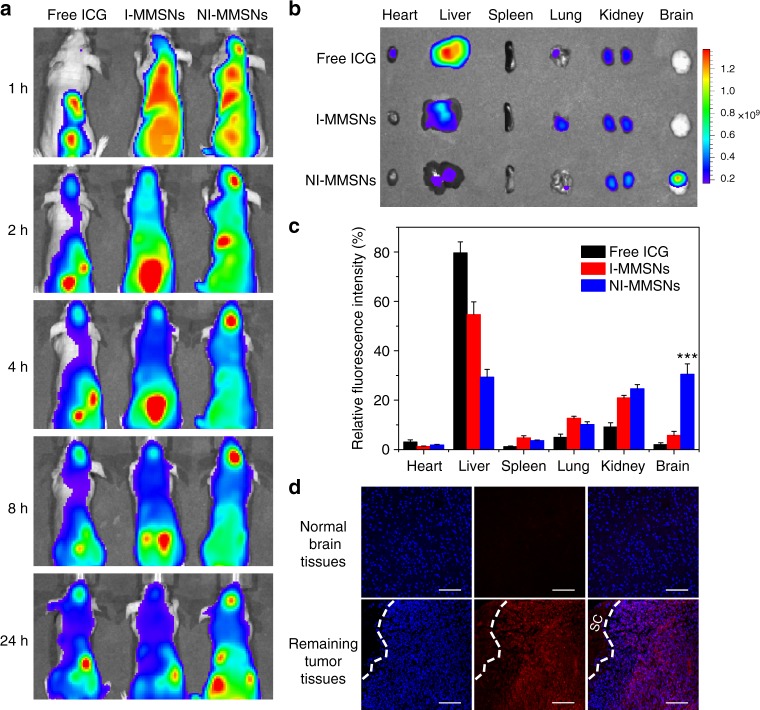
In vivo biodistribution of neutrophils carrying MMSNs. **a** In vivo fluorescence images of postsurgical glioma-bearing mice captured at the indicated time points after intravenous administration of free ICG, I-MMSNs, and NI-MMSNs. **b** Ex vivo fluorescence images of major organs excised from mice intravenously injected with free ICG, I-MMSNs, and NI-MMSNs at 4 h of postinjection. **c** Quantification analysis of relative fluorescence intensity in the organs at 4 h after intravenous injection. Mean values and error bars are defined as mean and s.d., respectively (*n* = 3). Statistical significance is assessed by Student’s two-sided *t* test compared to the control group. ***P < 0.001. **d** CLSM images of normal brain tissues and remaining tumor tissues in the postsurgical glioma-bearing mice after injection of NI-MMSNs. SC indicates the surgical cavity. Blue fluorescence: nuclei, red fluorescence: ICG. Scale bar: 100 μm

In vivo biodistribution of neutrophils after carrying theranostic MMSNs nanoparticles was further evaluated on the incomplete resection glioma model using an In Vivo Imaging System (IVIS Spectrum). MMSNs were fluorescently labeled with near-infrared dye indocyanine green (ICG) by physical entrapment (I-MMSNs), and then coincubated with neutrophils to prepare I-MMSN-internalized neutrophils (NI-MMSNs). As shown in Fig. 5a, NI-MMSNs reveal the efficient homing ability towards the inflamed brain tumor, emitting strong fluorescence at the surgically treated brain tumor only 1 h after injection. Much stronger fluorescence can be observed at the inflamed brain tumor site along with time till 24 h from the mice administrated with NI-MMSNs compared to that of mice injected with free ICG and I-MMSNs. To image and quantify the fluorescence signals in various organs, mice were euthanized at 4 h of postinjection, and the harvested organs were imaged (Fig. 5b). The results show that free ICG and I-MMSNs are mainly distributed in liver and kidney tissues. By contrast, the accumulation of NI-MMSNs in the liver is less than any other group, but significantly higher accumulation in the brain. The quantitative analysis reveals a fivefold increase of relative fluorescence intensity in the brain tumor in comparison with I-MMSNs, being consistent with that of in vivo fluorescence imaging (Fig. 5c).

Subsequently, the specific targeting of NI-MMSNs to the inflammatory brain sites was further determined by confocal laser scanning microscopy (CLSM) observation. As can be seen from Fig. 5d, the red fluorescence of ICG are largely merged with the blue fluorescence of remaining tumor tissues rather than normal brain tissues, illustrating that I-MMSNs could be efficiently delivered to the brain tumor lesions and penetrate the tumor foci, due to the inflammation-activatable homing of neutrophils. In addition, enhanced accumulation of NI-MMSNs in the inflamed brain tumor has been achieved by placing a magnet at the brain tumor area, showing a promising approach towards magnetic-guided immune cell therapy (Supplementary Fig. 11).

### In vivo therapeutic effect

**Fig. 6:**
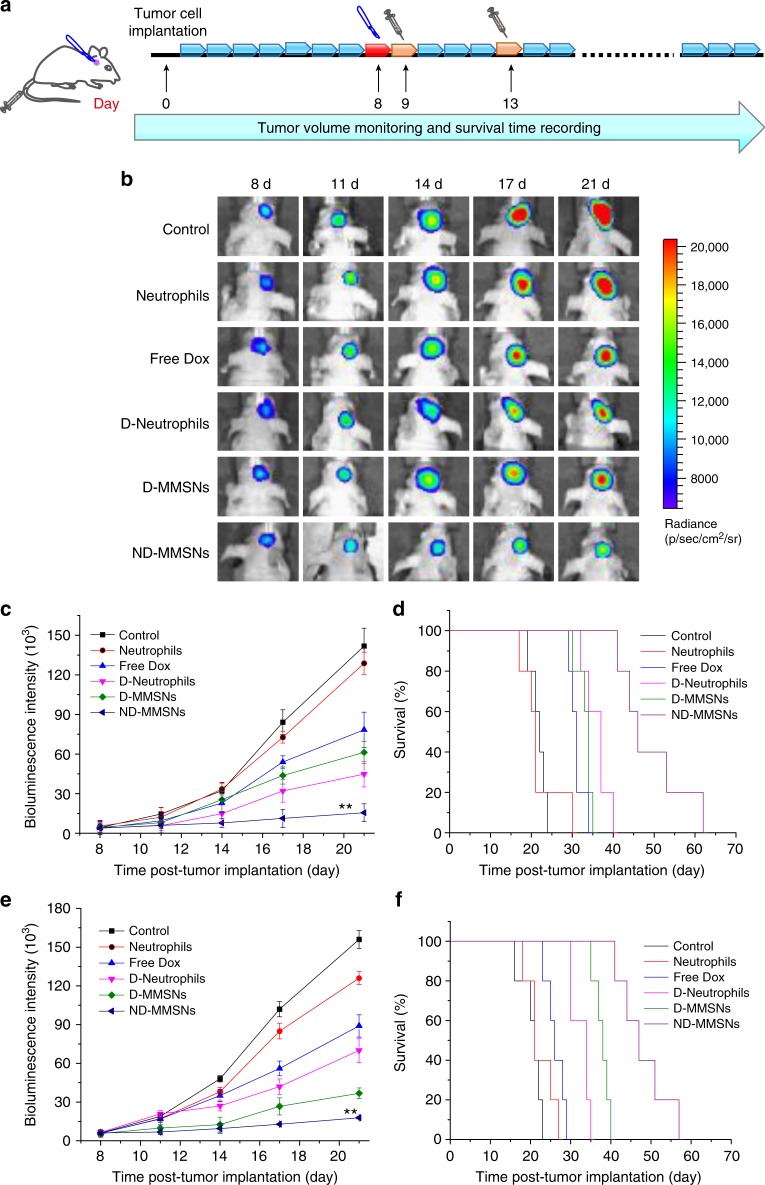
In vivo therapeutic performance of ND-MMSNs. **a** Schematic illustration of in vivo therapeutic schedule of mice after U87-Luc glioma cells implantation, primary glioma resection, and treatment of ND-MMSNs delivery system. **b** In vivo bioluminescent images, **c** quantified signal intensity and **d** survival curves of U87 glioma-bearing mice from each group after various treatment indicated. Statistical significance is assessed by Student’s two-sided *t* test compared to the control group. **P *<* 0.01. **e** Quantified signal intensity and **f** survival curves of C6 glioma-bearing mice from each group after various treatment indicated. Statistical significance is assessed by Student’s two-sided *t* test compared to the control group. **P < 0.01. Mean values and error bars are defined as mean and s.d., respectively (*n* = 5)

To further explore the therapeutic efficacy of ND-MMSNs, we applied them to an incomplete resection U87 glioma model that mimics postsurgical glioma recurrence. As can be seen from the in vivo therapeutic schedule, after incomplete surgery to remove part of primary glioma, the mice were randomly separated into six groups and intravenously administrated with saline (control), neutrophils alone, free Dox, Dox-internalized neutrophils (D-Neutrophils), D-MMSNs or ND-MMSNs (Fig. 6a). Then, the tumor volume was measured by monitoring the luminescence intensity via IVIS imaging and the survival time was recorded in the meantime. As shown in Fig. 6b−d, the bioluminescence intensity of brain tumor is similar in each group on the day of surgery. In addition, no significant difference is observed between control and neutrophils groups during the monitoring period. However, ND-MMSNs group exhibits the strongest inhibitory effect against tumor regrowth and the longest postsurgical survival time, suggesting the enhanced accumulation of Dox in the remaining glioma site due to the inflammation-triggered tumor targeting capability of neutrophils and the drug-encapsulating function of MMSNs. The median survival time of mice in ND-MMSNs group increases to 47 days compared with 23 days for control group, indicating systemic administration of ND-MMSNs after the resection of primary tumors could remarkably improve survival rate and delay glioma relapse. The incomplete resection C6 glioma model established in Balb/c nude mice confirms the therapeutic efficacy of neutrophil-based CDDSs again, showing the increased median survival time, enhanced tumor cell damage, and apoptosis in the ND-MMSNs group (Fig. 6e, f and Supplementary Fig. 12a, b).

### In vivo therapeutic mechanisms

**Fig. 7:**
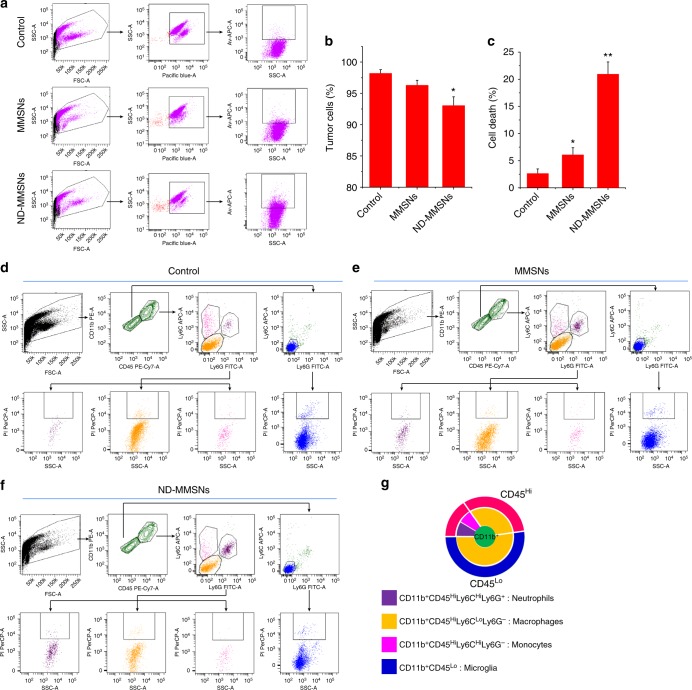
Flow cytometry evaluation of in vivo therapeutic mechanisms. **a**−**c** The single tumor cell suspension was stained by d-luciferin potassium salt and APC-labeled Annexin V apoptosis detection kit. The tumor cell ratio and the apoptotic tumor cells were determined by flow cytometry. Luciferin fluorescence that stands for tumor cells was detected with Pacific blue channel. Mean values and error bars are defined as mean and s.d., respectively (*n* = 3). Statistical significance is assessed by Student’s two-sided *t* test compared to the control group. *P < 0.05 and **P < 0.01. **d**−**f** Representative dot plots gated on the CD45^+^CD11b^+^ cells from tumors generated in U87 mice. The total population of CD45^+^CD11b^+^ cells is considered to be 100%, with CD11b^+^CD45^Hi^ (neutrophils, monocytes, and macrophages) and CD11b^+^CD45^lo^ (resident brain microglia) populations gated separately. Their expression of Ly6C and Ly6G was analyzed and the percentage of each subpopulation was quantified. The apoptotic cells in each subpopulation were also determined by PI. **g** Schematic diagram of tumor-associated macrophage ratio

For better understanding of the therapeutic mechanisms of ND-MMSNs, we performed flow cytometry to investigate the levels and status of tumor cells and tumor-associated macrophages (TAM). From Fig. 7a−c, we can see the number of tumor cells tends to decrease, from 98.17 ± 0.62% in untreated control mice to 93.03 ± 1.42% in ND-MMSN-treated mice. Apoptosis assay by flow cytometry reveals there are 20.92 ± 2.28 % apoptotic tumor cells in the ND-MMSN-treated mice, being significantly much more than that in the control and MMSN-treated mice. To characterize the TAM in murine brain tumors, we further employed standard antibody combinations for flow cytometric analysis. The CD45^+^CD11b^+^ myeloid cells in the ND-MMSN-treated brain tumors increase in comparison with MMSN-treated glioma and untreated control (Supplementary Fig. 13). Since CD11b^+^ myeloid cells can be subdivided into four distinct populations, including microglia (CD11b^+^CD45^Lo^), neutrophils (CD11b^+^CD45^Hi^Ly6C^Hi^Ly6G^+^), macrophages (CD11b^+^CD45^Hi^Ly6C^Lo^Ly6G^−^) and monocytes (CD11b^+^CD45^Hi^Ly6C^Hi^Ly6G^−^)^56^, we subdivided CD11b^+^ cells by using CD45, Ly6C, and Ly6G surface markers (Fig. 7d−g). As expected, the number of neutrophils increases in the MMSN-treated tumors due to surgical treatment, with a 3.03-fold higher than that of the untreated control. Interestingly, there is 7.61-fold higher neutrophils in the ND-MMSN-treated tumors, achieving 12.78 ± 1.42% cell ratio among the CD45^+^CD11b^+^ myeloid cells. Also, we find the macrophage ratio tends to decrease in the ND-MMSN-treated tumors (30.06 ± 2.36%), with 8.89 and 4.04% lower than that of the control and MMSN-treated tumors, respectively. Cell viability by flow cytometry assay reveals more neutrophils and macrophage died in the ND-MMSN-treated tumors (Supplementary Table 1). These results indicate neutrophils could really carry D-MMSNs and deliver into brain tumors after surgical treatment. Drug release from D-MMSNs would result in death of tumor cells and macrophage. Since TAM have been shown to play a major role in the creation of tumor microenvironment that promotes tumor progression, their apoptosis would greatly favor the antitumor efficacy^57^.

### Biosafety evaluation

In order to evaluate the biosafety of neutrophil-based theranostic platform for postsurgical glioma diagnosis and therapy, the mice were injected with MMSN-internalized neutrophils (N-MMSNs) to investigate the systemic toxic side effects. Their major organs were harvested for hematoxylin and eosin (H&E) pathological staining. Compared to saline group, there is no noticeable pathological abnormalities or inflamed cells in heart, liver, spleen, lung, and kidney in the treatment group, suggesting no organ damage or inflammatory lesion occurs after N-MMSNs treatment (Supplementary Fig. 14).

## Discussion

An intelligent biomimetic theranostic platform has been developed by integrating inflammation-activatable neutrophils with core-shell structured MMSNs for treating remaining glioma after surgical resection of primary tumors. ND-MMSNs were prepared by internalizing Dox-loaded MMSNs into neutrophils, which still remained a cellular activity and could actively target the inflamed glioma site for therapeutic purpose. Moreover, the phagocytic MMSNs possessed cell tracking capability, providing the potential for the diagnosis of residual tumor and therapeutic guidance. Importantly, improved survival rate and delayed glioma relapse were demonstrated in the surgically treated glioma mouse models after treatment with ND-MMSNs, suggesting that neutrophils carrying D-MMSNs could effectively identify the inflammatory signals derived from surgical management and accumulate in the remaining tumor site to maximize the drug bioavailability. This strategy provides a new insight to track the fate of neutrophils by MRI and explore immune CDDSs for treating diseases associated with inflammation.

## Methods

### Materials and reagents

Oleic acid-capped Fe_3_O_4_ nanoparticles, synthesized by a traditional high temperature pyrolysis method, were obtained from Nanjing Nanoeast Biotech Co. Ltd. Tetraethyl orthosilicate (TEOS), CTAB, Percoll, and FITC were purchased from Sigma-Aldrich. Sodium hydroxide (NaOH), chloroform, and ethanol were obtained from Sinopharm Chemical Reagent Co. Cell counting kit-8 (CCK-8) was purchased from Dojindo Molecular Technologies, Inc. 4′,6-diamidino-2-phenylindole (DAPI) staining solution was bought from Beyotime Institute of Biotechnology. Calcein acetoxymethyl ester (calcein-AM) and propidium iodide (PI) were purchased from Thermo Fisher Scientific Inc. Mouse TNF-α ELISA kit and IL-6 ELISA kit were purchased from eBioscience. All chemicals were used as received without further purification, and their aqueous solutions were prepared using deionized water.

### Preparation of MMSNs

0.5 g of CTAB was added into 20 ml of deionized water, followed by adding 0.8 ml of oleic acid-capped Fe_3_O_4_ nanoparticles dispersed in chloroform solution (5 mg ml^−1^). The mixture was stirred vigorously for 40 min and then the temperature was raised to 60^o^ C to evaporate the chloroform, resulting in a transparent black Fe_3_O_4_/CTAB solution. Subsequently, the solution was added into a mixture of 25 ml of deionized water and 0.15 ml 2 M of NaOH and the temperature was raised to 70° C under stirring. Then, 0.25 ml of TEOS and 1.5 ml of ethyl acetate were added into the above solution in sequence and the reaction was continued for 3 h. The material was collected by centrifugation and washing with ethanol for several times. Finally, the template CTAB was extracted by dispersing the as-synthesized material in the ethanolic ammonium nitrate (10 mg ml^−1^) at 60 °C for 1.5 h. The product was then centrifuged and washed with ethanol.

### Characterizations

TEM images and energy dispersive X-ray spectrum were acquired using a JEM-2100F transmission electron microscope. Element mapping was captured by FEI Magellan 400 scanning electron microscope (USA). N_2_ adsorption−desorption isotherm was measured on a Micrometitics Tristar 3000 system. DLS measurement and Zeta potential were conducted on a Zetasizer Nanoseries instrument (Nano ZS90). The magnetization curve was obtained at room temperature under a varying magnetic field using a Vibrating Sample Magnetometer (VSM, PPMS Model 6000 Quantum Design). The concentrations of silicon and iron were measured by using inductively coupled plasma optical emission spectrometry (ICP-OES, OPTIMA 7000DV).

### Isolation of neutrophils

Percoll gradient method was used for the isolation of neutrophils from peripheral blood of mice. Briefly, blood samples were collected in tubes with 3.8% sodium citrate, purified by centrifugation and then resuspended in 2 ml of PBS. The cell pellets were then carefully added into a three-layer Percoll gradient of 78, 69, and 52% (v/v) diluted in PBS, followed by centrifuging at 2000 rpm for 20 min at room temperature. The neutrophils were recovered from the 69 and 78% interface and the upper part of the 78% layer. After the lysis of residual erythrocytes by lysis buffer at 4 °C, neutrophils with high purity were obtained.

### Preparation of ND-MMSNs

Firstly, Dox-loaded MMSNs (D-MMSNs) were prepared by dispersing 20 mg of MMSNs into 20 ml of Dox PBS solution (0.5 mg ml^−1^) and stirring at room temperature under dark conditions for 24 h. Then, D-MMSNs were collected by centrifugation and freeze-drying under vacuum. The Dox concentration in the supernatant solution was measured by UV−Vis spectra at *λ* = 480 nm. The Dox loading efficiency can be calculated as follows: Dox loading efficiency = (initial Dox - supernatant Dox)/initial Dox.

Secondly, ND-MMSNs were obtained by incubating isolated neutrophils with D-MMSNs at a Dox concentration of 10 μg ml^−1^ at 37 °C in fresh serum-free media for 1 h. After centrifuging and washing with ice-cold PBS twice, ND-MMSNs suspension were obtained. The uptake of D-MMSNs by neutrophils was determined by flow cytometry (BD FACSCanto, BD Biosciences).

### In vitro Dox and iron release

5 mg of D-MMSNs were packaged into a dialysis bag with a cutoff molecular weight of 8 kDa and immersed into 30 ml of releasing media with different pH values (pH = 7.4, 6.0 or 5.0) in a tube, which were further performed on a shaking table with the shaking speed of 100 rpm at 37 °C. At different time points, 3 ml of releasing solution was collected and measured by using UV−Vis absorbance spectrometer (*λ* = 480 nm), and then added 3 ml of fresh releasing media back to the tube. The concentration of iron was quantified by ICP-OES after the UV−Vis absorbance spectrometer test.

### Flow cytometry evaluation of D-MMSNs uptake by neutrophils

1×10^5^ neutrophils were treated with culture media and D-MMSNs ([Dox] = 10 μg ml^−1^, 0.5 ml) for 1 h at 37 °C. At the end of incubation, the neutrophils were washed with ice-cold PBS thrice, trypsinized and resuspended in the media. The intracellular Dox fluorescence was determined by BD Biosciences FACSCanto flow cytometer via the PE-A channel.

### Assessment of the cytotoxicity of MMSNs or D-MMSNs against neutrophils

The neutrophils were seeded in a 96-well culture plate at a density of 5×10^3^ per well. Then, the cells were incubated with MMSNs at different concentrations or D-MMSNs at various Dox concentrations in 5% CO_2_ at 37 °C for 1, 2, and 12 h. At the end of incubation, the cell media were removed and replaced with CCK-8 (100 μl, *V*_CCK 8_:*V*_DMEM_ = 1:9, DMEM means Dulbecco's Modified Eagle Media). After that, the absorbance was measured on a microplate reader at the wavelength of 450 nm. The relative cell viability was calculated as (*A*_t_ − *A*_nc_)/(*A*_pc_ − *A*_nc_) × 100%, where *A*_t_, *A*_pc_, and *A*_nc_ indicate the absorbance of tested groups, positive, and negative controls, respectively.

### Fluorescent live/dead cells staining experiment

The neutrophils were coincubated with D-MMSNs ([Dox] = 10 μg ml^−1^) in 5% CO_2_ at 37 °C for 0, 1, 2, and 12 h. At the end of incubation, the culture media were removed and replaced with 100 μl of calcein-AM and 100 μl of PI solution for 15 min. Finally, the cells were observed by fluorescence micoroscope, where live cells were stained in green and dead cells in red, respectively.

### T_2_-MR imaging in vitro

The D-MMSNs (0, 12.5, 25, and 50 μg ml^−1^) were incubated with neutrophils (5×10^6^ per tube) in 5% CO_2_ at 37 °C. After coincubation for 1 h, the cells were washed with PBS for three times, and neutrophils carrying D-MMSNs in PBS were precipitated at the bottom of tube after centrifugation. The MRI experiments were performed on a 3.0 T clinical MRI instrument.

### Cell culture

Microvascular endothelial cell line bEnd.3, human glioma cell line U87, and rat glioma cell line C6 were purchased from American Type Culture Collection, and are not listed by International Cell Line Authentication Committee as cross-contaminated or misidentified cell lines (v8.0, 2016). All these cells were cultured in DMEM supplemented with 10% fetal bovine serum (FBS), 100 mg ml^−1^ streptomycin and 100 units ml^−1^ penicillin, and cultured at 37 °C in a humidified atmosphere with 5% CO_2_. Notably, throughout the studies, all cells were tested negative for mycoplasma contamination.

### Intercellular delivery of drug from neutrophils to glioma cells

In order to track the intercellular transport of drug, MMSNs were labeled with FITC. First, 15 mg of FITC was dispersed into 5 ml of ethanol, followed by adding 100 μl of APTES. The mixture was stirred under dark conditions for 24 h. Subsequently, 20 mg of MMSNs were reacted with 0.5 ml of FITC-APTES stock solution under dark conditions for another 24 h. FITC-labeled MMSNs (FMMSNs) were collected by centrifugation and washed with ethanol for several times until no FITC can be detected in ethanol solution. Dox-loaded FMMSNs (D-FMMSNs) were prepared according to the previous procedure in the section of preparation of ND-MMSNs.

The intercellular delivery of drug was observed by using CLSM. 2×10^4^ U87 or C6 glioma cells were seeded in the confocal dishes and cultured overnight. Then, neutrophils carrying D-FMMSNs with Dox concentration of 10 μg ml^−1^ were added into the seeded cells and coincubated for 2 or 4 h. At the end of incubation, the cells were washed with PBS three times, fixed with 4% paraformaldehyde solution for 20 min and stained with DAPI for 15 min, followed by observation under CLSM (Leica TCS SP5).

### Cytotoxicity of neutrophils or ND-MMSNs against U87, C6, or bEnd.3 cells

The U87, C6, or bEnd.3 cells were seeded in a 96-well culture plate at a density of 5×10^3^ per well and cultured overnight. Then, the cells were incubated with neutrophils of different numbers (625, 1250, 2500, 5000, 10,000, 20,000, and 40,000) or ND-MMSNs at varied Dox concentrations (0.625, 1.25, 2.5, 5, 10, 20, and 40 μg ml^−1^) in 5% CO_2_ at 37 °C for 24 h. At the end of incubation, the cell media were removed and replaced with CCK-8 (100 μl, *V*_CCK 8_:*V*_DMEM_ = 1:9). Finally, the measurement of absorbance and calculation of relative cell viability were processed according to the previous method in the section of assessment of the cytotoxicity of MMSNs or D-MMSNs against neutrophils.

### Chemotactic migration in vitro

For chemotactic migration assay, bEnd.3 cells were seeded on the lower chamber of six-well transwell plates (3 μm pore size, Corning Incorporated). After incubation for 12 h, the bEnd.3 cells were exposed to fresh serum-free media with 20 ng ml^−1^ of TNF-α and these groups were used as treatment groups. The bEnd.3 cells exposing to fresh serum-free media were used as control group. After 4 h, 1 ml of ND-MMSNs (1×10^6^ cells ml^−1^) suspending in fresh serum-free media were placed in the upper compartment. After incubating at 37 °C in a 5% CO_2_-humidified incubator for different time, nonmigrated cells on the filter membrane were removed using a cotton swab, whereas the cells that migrated to the lower membrane surface were fixed, stained with 0.1% crystal violet and viewed by light microscope.

### Animal model

Six- to eight-week-old female Balb/c nude mice (18–22 g) were purchased from the Medical Experimental Animal Center of Guangdong Province. All animal experiments were performed under the guidelines approved by the Animal Study Committee of Shenzhen Institutes of Advanced Technology, Chinese Academy of Sciences.

To establish an incomplete resection glioma model, the nude mice were intracranially injected with 5×10^5^ U87-Luc or C6-Luc cells in 5 μl PBS on the right hemisphere of the brain using a stereotactic fixation device. The brain tumor growth was monitored by intraperitoneally injecting the d-luciferin potassium salt (150 mg kg^−1^) and observing the luminescence intensity of U87-Luc or C6-Luc cells in the brain using an In Vivo Imaging System (Caliper IVIS Spectrum). At 8 (U87-Luc cells) or 9 days (C6-Luc cells) after glioma cells were transplanted into the brain of mice, tumors were surgically resected leaving residual tissue of similar volume behind. Less than 5% mortality was observed owing to the surgical procedure.

### T_2_-MR imaging in vivo

In vivo MR imaging was conducted on a clinical MRI (Siemens Magnetom Trio, 3.0 T) scanner. The postsurgical glioma-bearing mice were randomly assigned to two groups and anesthetized with 1–2% isoflurane in 20% oxygen. T_2_-weighted MRI of mice were collected before and 0.5, 1, 1.5, and 2 h after administration of D-MMSNs and ND-MMSNs (1×10^6^ neutrophils per mouse) with a dose of 150 μl (3 mg kg^−1^ Fe) via intravenous injection. T_2_-weighted MRI of brain sections were performed with a fast spin echo sequence: TR = 5000 ms, TE = 79.2 ms, slice thickness = 1.5 mm, FoV = 40 × 40. The signal intensities of regions of interest in the brain tumor were measured before and after injection. Accumulation of contrast agents within the tumor was further confirmed by ex vivo Prussian blue staining images of the tumor tissues after 2 h of intravenous injection.

### In vivo biodistribution of neutrophils carrying MMSNs

The surgically treated glioma-bearing mice were randomly grouped and administrated with free ICG, I-MMSNs, and NI-MMSNs (1×10^6^ cells per mouse, 5 mg kg^−^^1^ ICG) via tail vein, respectively. Subsequently, the mice were anesthetized with 1–2% isoflurane in 20% oxygen and imaged at 1, 2, 4, 8, and 24 h post administration using the IVIS Spectrum imaging system. For ex vivo fluorescence imaging, the brains and other organs were also collected, rinsed and imaged at 4 h of postinjection. Moreover, the brain tissues were frozen in cryoembedding media (OCT) at −80 °C and cut into 20 μm sections. After staining with DAPI, the slides were observed under a confocal laser scanning microscope (Leica, Germany).

### In vivo therapeutic effect

The surgically treated glioma-bearing mice were randomly divided into six different groups that received intravenous injection as following: (1) saline; (2) neutrophils (1×10^6^ cells per mouse); (3) free Dox (5 mg kg^–^^1^ Dox); (4) D-Neutrophils (1×10^6^ cells per mouse, 5 mg kg^−1^ Dox); (5) D-MMSNs (5 mg kg^−1^ Dox); (6) DN-MMSNs (1×10^6^ cells per mouse, 5 mg kg^−1^ Dox) at 1 and 5 d after surgery. To monitor the tumor progression, the bioluminescence images were measured at different time intervals after the injection. In addition, the survival time of each group was recorded.

### Flow cytometry evaluation of in vivo therapeutic mechanisms

Brain tumors from untreated control, MMSNs- or ND-MMSNs-treated 48 h were digested in Collagenase IV (400 U ml^−1^) and Dispase (1.2 U ml^−1^) and DNase I (32 U ml^−1^) in DPBS with Mg^2+^/Ca^2+^ at 37 °C for about 45 min. Digestion was terminated by adding 1 ml FBS and 20 ml cold PBS. Cells were passed through a 40 μm cell strainer, centrifuged and resuspended in staining buffer (3 % FBS/PBS). As for determination of the tumor cells, the single cell suspension was stained by d-luciferin potassium salt and APC-labeled Annexin V apoptosis detection kit. The tumor cell ratio and the apoptotic tumor cells were determined by flow cytometry. As for determination of the myeloid cells, the cells were stained using the following antibodies: PE/Cy7®-conjugated CD45 antibody (Abcam, ab210186), allophycocyanin-conjugated Ly6c antibody (Abcam, ab93550), FITC-conjugated Ly6g antibody (Abcam, ab210203), and phycoerythrin-conjugated CD11b antibody (Abcam, ab25175).

### In vivo safety evaluation

Healthy Balb/c mice with average weight of 20 g were intravenously injected with neutrophils carrying MMSNs (1×10^6^ cells per mouse) and this group of mice was used as the experimental group (*n* = 5). The mice received with saline were selected as the control group. The major organs from the control and experimental groups were harvested, fixed in paraformaldehyde in PBS (4 %) and stained with H&E at 30 d after injection. The histological sections were observed under an optical microscope.

### Statistical analysis

Mean values and error bars are defined as mean and s.d. ^*^P < 0.05, ^**^P < 0.01 and ^***^P < 0.001. The samples/animals were allocated to experimental groups and processed randomly.

## Electronic supplementary material

Supplementary Information

## Data Availability

All data are available from the authors upon reasonable request.
