# Supplementary material for: MR imaging tracking of inflammation-activatable engineered neutrophils for targeted therapy of surgically treated glioma
Source: Nat Commun. 2018 Nov 14;9:4777. doi: 10.1038/s41467-018-07250-6 (PMC6235838; doi:10.1038/s41467-018-07250-6)
Supplement: Supplementary file 1 — Supplementary Information [file 41467_2018_7250_MOESM1_ESM.pdf]

Supporting information

MR imaging tracking of inflammation-activatable engineered neutrophils for targeted therapy of surgically treated glioma

Wu et al.

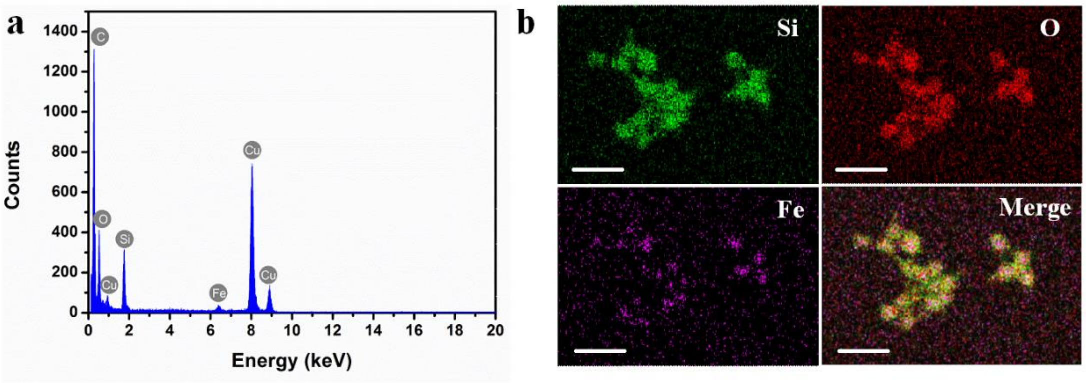

Supplementary Figure 1. (a) Energy dispersive X-ray spectrum of MMSNs. (b) Element mapping of Si, O, Fe elements in the MMSNs. Scale bar: 100 nm.

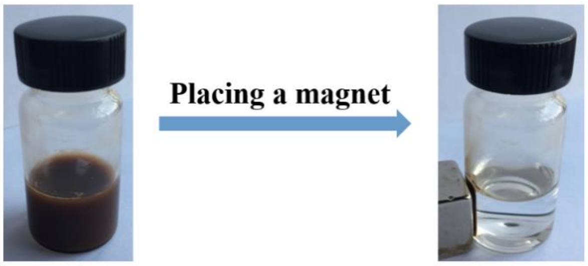

Supplementary Figure 2. Photographs of MMSNs well-dispersed in aqueous solution (left) and the collection of MMSNs from aqueous solution with a magnet (right).

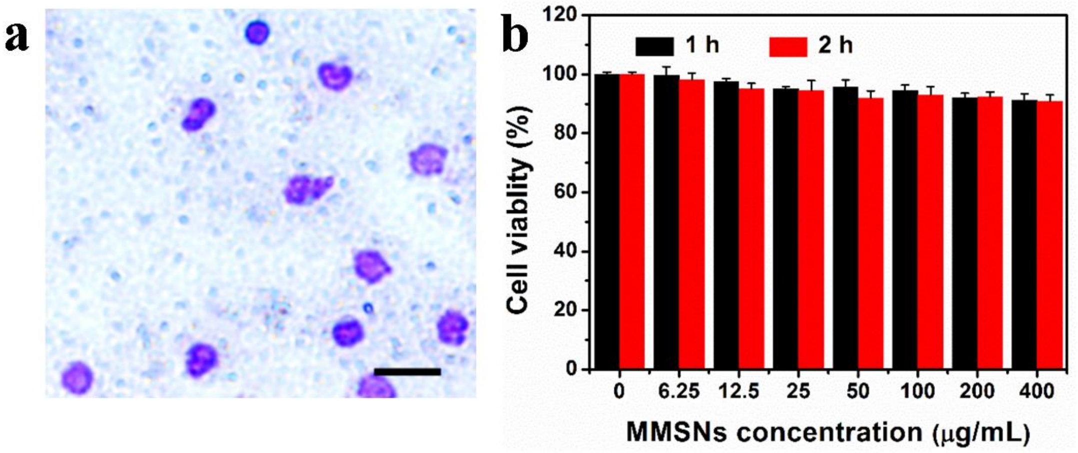

Supplementary Figure 3. (a) Optical microscope image of isolated neutrophils stained with Wright-Giemsa. Scale bar: 20 μm. (b) Cell viabilities of neutrophils incubated with MMSNs at different concentrations in 1 and 2 h.

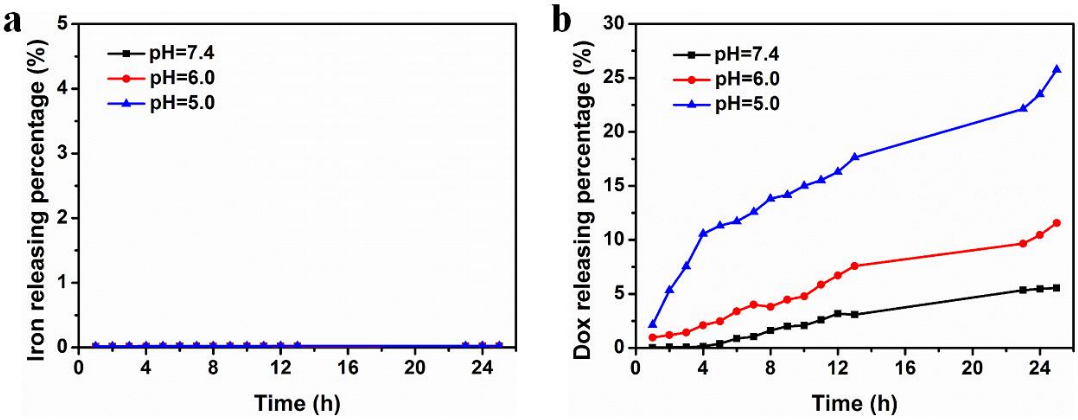

Supplementary Figure 4. (a) Iron releasing percentage and (b) Dox releasing percentage of D-MMSNs under different pH buffer solutions (pH = 7.4, 6.0 or 5.0).

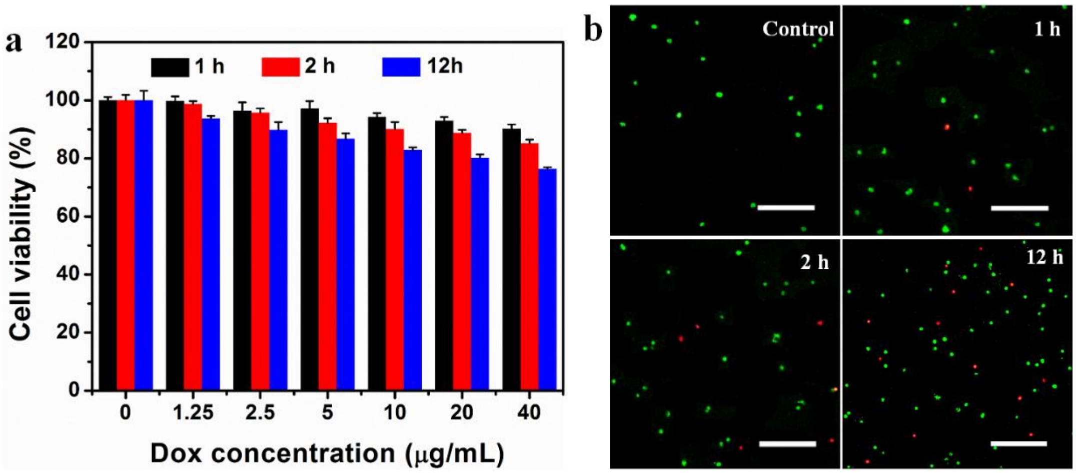

Supplementary Figure 5. (a) Cell viabilities of neutrophils incubated with D-MMSNs at varied Dox concentrations in 1, 2 and 12 h. (b) Fluorescence microscopy images of neutrophils treated with D-MMSNs for 0, 1, 2 and 12 h, respectively ([Dox] = 10 μg ml^{-1}). Live and dead cells were stained by Calcein AM and PI, and presented in green and red colors in those images, respectively. Scale bar: 100 μm.

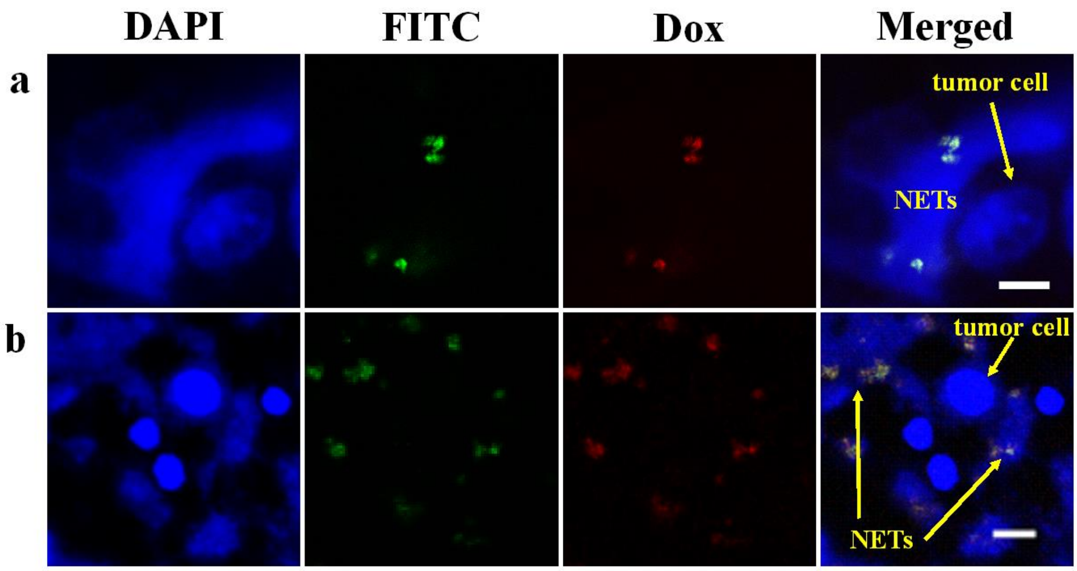

Supplementary Figure 6. CLSM images of (a) U87 or (b) C6 glioma cells incubated with ND-FMMSNs for 2 h under high and low magnification. Glioma cell nuclei and neutrophil-derived DNA networks were stained with DAPI emitting blue fluorescence. Green fluorescence: FITC-labeled MMSNs. Red fluorescence: Dox. (a) Scale bar: 5 μm. (b) Scale bar: 10 μm.

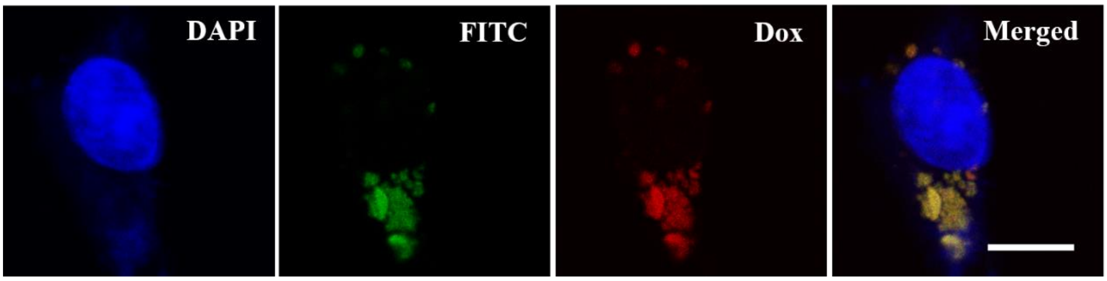

Supplementary Figure 7. CLSM images of C6 glioma cells after incubation with ND-FMMSNs for 4 h ([Dox] = 10 μg ml^{-1}). After incubation, C6 cell nuclei were stained with DAPI emitting blue fluorescence. Green fluorescence: FITC-labeled MMSNs. Red fluorescence: Dox. Scale bar: 10 μm.

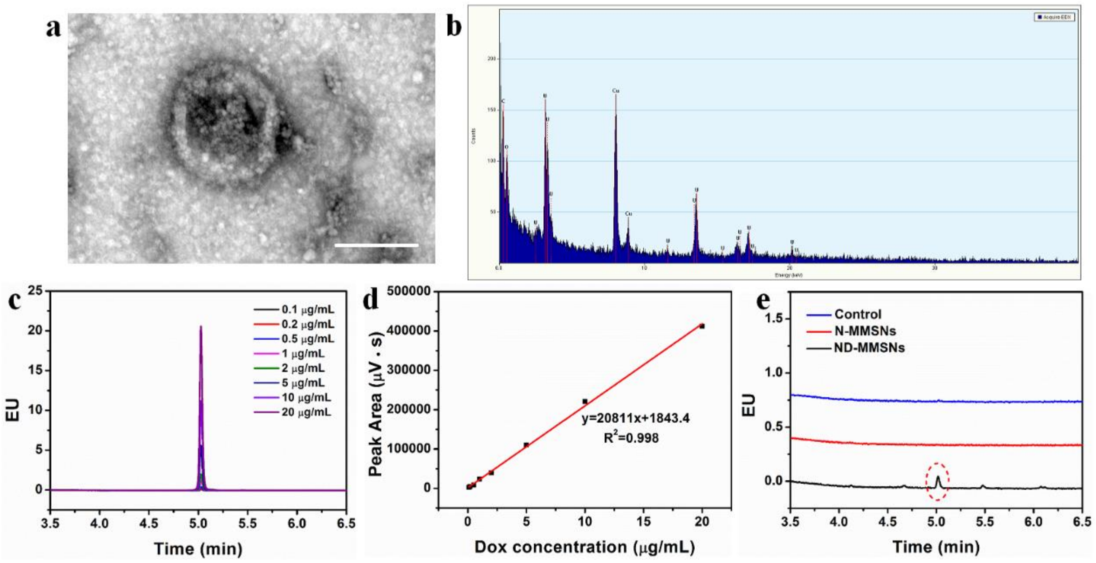

Supplementary Figure 8. (a) TEM image and (b) energy dispersive X-ray spectrum of ND-MMSN-derived exosomes by employing density gradient centrifugation. Scale bar: 200 nm. (c) HPLC chromatograms of different Dox concentration solutions. (d) The linear relationship between peak area and Dox concentration. (e) HPLC chromatograms of neutrophil derived exosomes, N-MMSN-derived exosomes and ND-MMSN-derived exosomes.

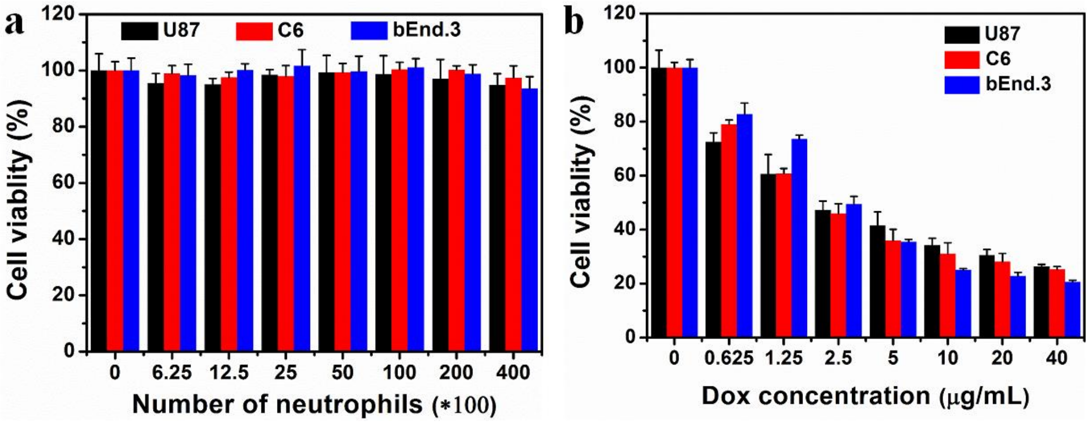

Supplementary Figure 9. Cell viabilities of U87, C6 or bEnd.3 cells incubated with (a) neutrophils of different numbers or (b) ND-MMSNs at varied Dox concentrations in 24 h.

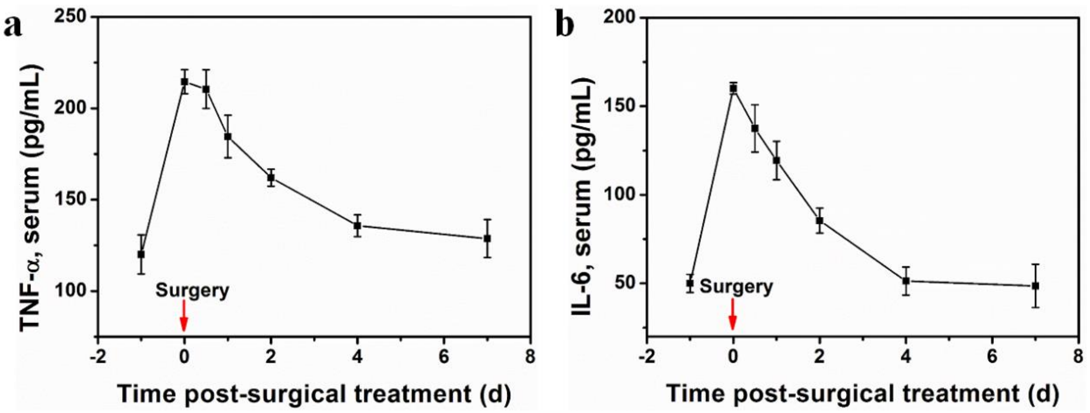

Supplementary Figure 10. Determination of the inflammation cytokines TNF-α (a) and IL-6 (b) levels in the serum of the U87-bearing mice after surgical treatment for 7 d. Mean values and error bars are defined as mean and s.d., respectively (n = 3).

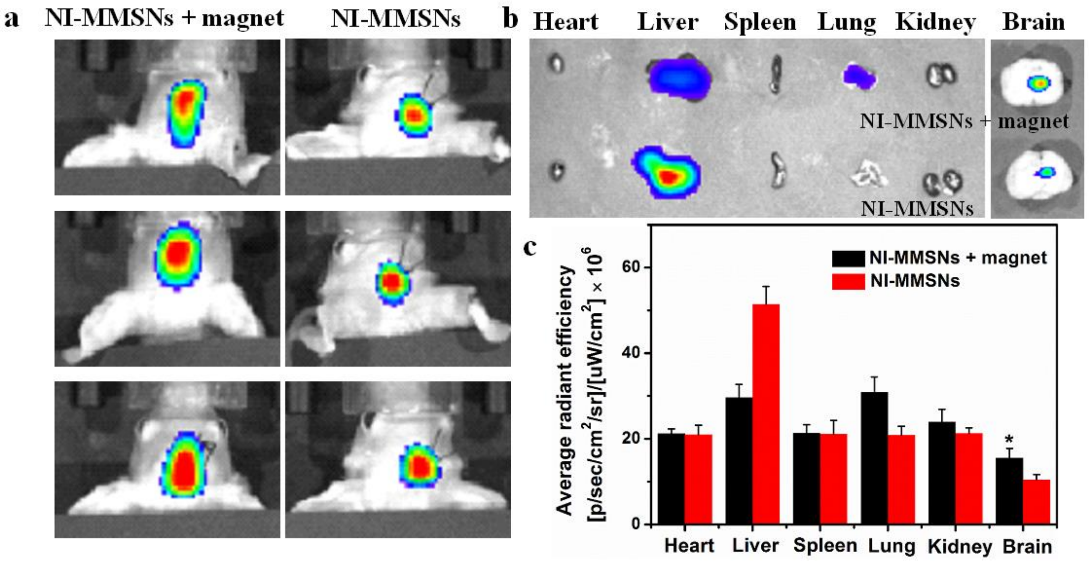

Supplementary Figure 11. (a) In vivo fluorescence images of postsurgical glioma-bearing nude mice after intravenous injection of NI-MMSNs with or without magnetic field. (b) Ex vivo fluorescence images of major organs and brain harvested at 4 h of postinjection. (c) The average fluorescence signal intensities in different groups. Mean values and error bars are defined as mean and s.d., respectively (n = 3). Student's two-sided *t* test.$^{*}P < 0.05$.

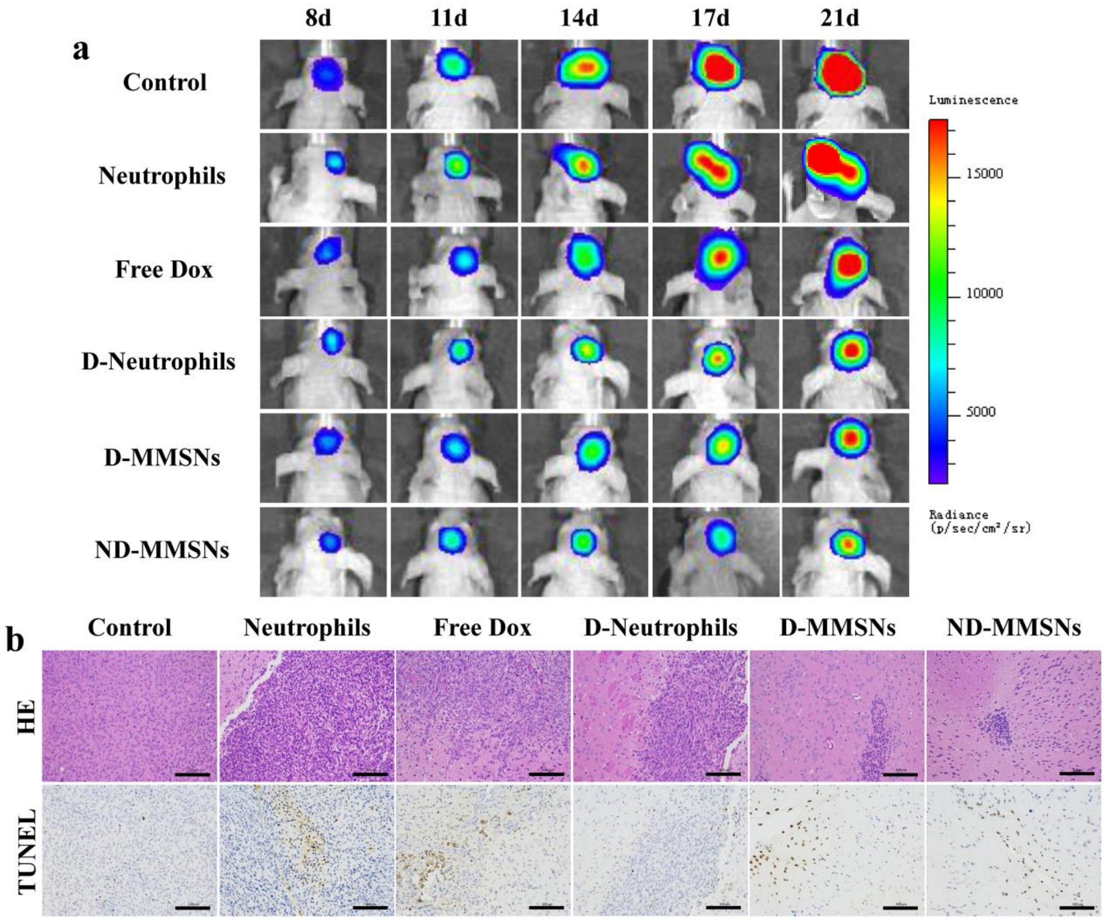

Supplementary Figure 12. (a) In vivo bioluminescent images of C6 glioma-bearing mice from each group after various treatment indicated. (b) Histopathology images of H&E-stained and TUNEL-stained tumor slices collected from mice 2 d after various treatments. Scale bar: 100 μm.

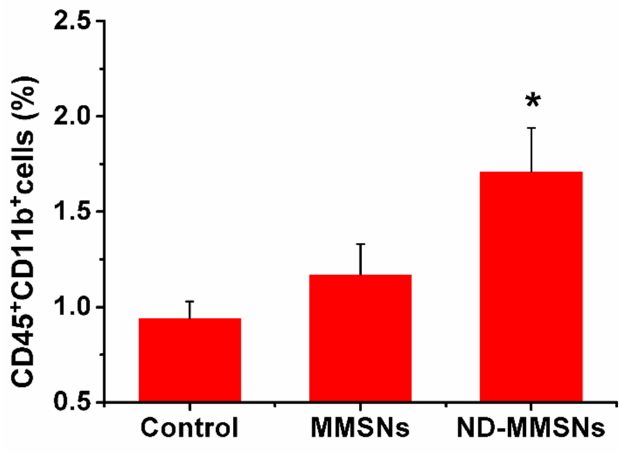

Supplementary Figure 13. The percentage of $CD45^{+}CD11b^{+}$ cells in all of the cells. Mean values and error bars are defined as mean and s.d., respectively (n = 3). Student's two-sided *t* test. $^{*}P < 0.05$.

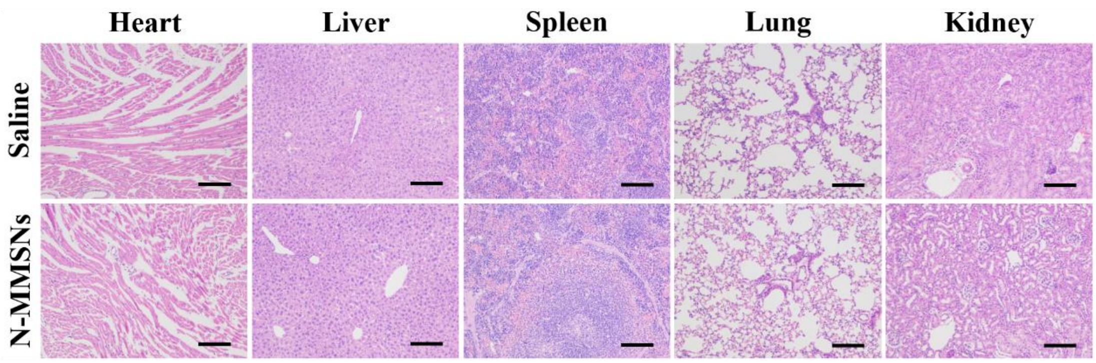

Supplementary Figure 14. Histological examinations of the major organs (heart, liver, spleen, lung and kidney) from mice after treatment with saline and N-MMSNs. Scale bar: 100 μm.

Supplementary Table 1. The cell ratio and apoptotic cell ratio in $CD45^+CD11b^+$ myeloid cells.

Group	Myeloid cells	Cell ratio (%)	Apoptotic ratio (%)
Control	Neutrophils	1.68 ± 0.62	2.82 ± 0.86
	Macrophages	38.95±2.78	2.06 ±0.38
	Macrocytes	3.54 ± 0.35	4.51 ± 0.28
	Microglia	55.82 ±2.16	4.19 ±0.96
MMSNs	Neutrophils	5.09 ± 0.84*	3.72 ± 1.34
	Macrophages	34.10 ±1.42	3.40 ± 0.63
	Macrocytes	2.21 ± 0.85	4.02 ± 0.84
	Microglia	58.60 ±2.47	2.30 ± 0.46
ND-MMSNs	Neutrophils	12.78 ± 1.42**	7.57 ± 1.32*
	Macrophages	30.06 ±2.36*	6.71 ± 1.37*
	Macrocytes	2.94 ± 0.58	3.50 ± 0.71
	Microglia	54.22 ±2.86	3.18 ± 0.82

Note: The total population of $CD45^+CD11b^+$ cells is considered to be 100 %. Mean values and error bars are defined as mean and s.d., respectively (n = 3). Student's two-sided *t* test. $^{*}P < 0.05$ and $^{**}P < 0.01$.
